# The Rubber Suits

**Published:** 1868-03

**Authors:** S. S. Fisher


### THE RUBBER SUITS.

We give below some extracts from the Briefs of the argument of Defendant's counsel, Col. S. S. Fisher, in the cases of the Dental Vulcanite Co., against Dr. A. Berry, of Cincinnati, and E. Honsinger, of Chicago, in the Circuit Courts; argued in February last, which will give a very clear idea of the points made, and the line of argument, at least so far as it pertains to the patents under which the Dental Vulcanite Co. are operating.

The remaining part of the argument presents very forcibly the fact that the defendants are now, and have been for some time using a compound, different in character from that prepared under the Goodyear patent, and for which a patent has been granted to E. L. Simpson, of Bridgeport, Conn.

No decision has yet been rendered in these cases.—Ed.

---

*Circuit Court of the United States, Southern District of Ohio. Henry B. Goodyear*, vs. *Archibald Berry—Brief for Defendant.*

I. *The two reissued patents upon which this suit is brought are void, because they are not for the same invention as the original.*

The original patent describes a compound of India rubber with sulphur and certain other substances named, which compound is subjected to a high degree of heat, in accordance with the process of Charles Goodyear. No other gum than India rubber is mentioned in the specification or claim, nor is there any hint that the process can be applied to any other gum, or that any other gum is vulcanizable. The entire specification is found on p. 327 of defendants' New York testimony. The claims are as follows:

"What I do claim as my invention, and desire to secure by letters patent, is the combining of India rubber and sulphur, either with or without shellac, for making a hard and inflexible substance hitherto unknown, substantially as herein set forth."

"And I also claim 'the combining of India rubber, sulphur, and magnesia or lime, or a carbonate or a sulphate of magnesia or of lime, either with or without shellac, for making a hard and inflexible substance, hitherto unknown, substantially as herein set forth.' "

The reissued patents interpolate the words "other vulcanizable gums" and "other allied gums" after every mention of India rubber or caoutchouc. The claims of the reissues are as follows:

"Reissue No. 556.

"But what I do claim as the invention of the said Nelson Goodyear, and desire to secure by letters patent, is the combining of sulphur and India rubber, *or other vulcanizable gum*, in proportion substantially as specified, when the same is subjected to a high degree of heat, substantially as specified, according to the vulcanizing process of Charles Goodyear, for the purpose of producing a substance or manufacture possessing the properties or qualities substantially such as described; and this I claim whether the said compound of sulphur and gum be, or be not, mixed with other ingredients, as set forth.

"Reissue No. 557.

"What is claimed as the invention of the said Nelson Goodyear, deceased, and desired to be secured by letters patent, is the new manufacture or substance herein above described, and possessing the substantial properties herein described, and composed of India rubber, *or other vulcanizable gum*, and sulphur, in the proportions substantially such as described, and when incorporated, subjected to a high degree of heat, as set forth; and this I claim whether other ingredients be, or be not, used in the preparation of said manufacture, as herein described."

Interpolations into the specification and claim, of matters not found in the original specification or drawings, avoid the patent.

*Carhart* v. *Austin*, 2 Fisher, 543.
*Sickles* v. *Evans*, 2 Fisher, 437.

Although the Patent Office at one time seemed to suppose that proof, outside of the record, might be received to show what the patentee's invention was, yet this was so obviously improper that the present rule expressly forbids it. Rule 52, Edit. 1867, provides as follows:

"52. The general rule is, that whatever is really embraced in the original invention, and so described or shown that it might have been embraced in the original patent, may be the subject of a reissue, *but an applicant will not be allowed the benefit of proof that there was more in his invention than is shown in his original application, model or specimens.*"

The claim for "other vulcanizable gum" is not only broader than the original invention, but if it had been appended to the original patent that patent would have been void, because an inventor has no right to claim a process as applied to "all other vulcanizable gums." He has not discovered that all other gums can be subjected to his process. He does not name any such gums, nor describe how his process is to be applied to them. He claims the unknown and the undiscovered, as well as the known, that he has no right to throw the pall of his monopoly over the future discoveries and inventions of other men.

*O'Reilly* v. *Morse*, 15 How. 112–119.
*Leroy* v. *Tatham*, 14 How. 175.
*Sickles* v. *Gloucester Co.*, 1 Fisher, 238.
*Sickles* v. *Evans*, 2 Fisher, 435–438.
*Burr* v. *Duryee*, 1 Wall. 576, 577.

In *O'Reilly* v. *Morse*, p. 113, the Supreme Court say:

"Nor is this all. While he shuts the door against inventions of other persons, the patentee would be able to avail himself of new discoveries in the properties and powers of electro-magnetism, which scientific men might bring to light. For he says he does not confine his claim to the machinery or parts of machinery which he specifies; but claims for himself a monopoly in its use, however developed, for the purpose of printing at a distance. New discoveries in physical science may enable him to combine it with new agents and new elements, and by that means attain the object in a manner superior to the present process, and altogether different from it. And if he can secure the exclusive use by his present patent he may vary it with every new discovery and development of the science, and need place no description of the new manner, process, or machinery upon the records of the Patent Office. And when his patent expires, the public must apply to him to learn what it is. In fine, he claims an exclusive right to use a manner and process which he has not described, and, indeed, had not invented, and, therefore, could not describe when he obtained his patent.

"The Court is of opinion that the claim is too broad, and not warranted by law.

"No one, we suppose, will maintain that Fulton could have taken out a patent for his invention of propelling vessels by steam, describing the process and machinery he used, and claimed under it the exclusive right to use the motive power of steam, however developed, for the purpose of propelling vessels. It can hardly be supposed that under such a patent he could have prevented the use of the improved machinery which science has since introduced; although the motive power is steam, and the result is the propulsion of vessels.

"Neither could the man who first discovered that steam might, by a proper arrangement of machinery, be used as a motive power to grind corn, or spin cotton, claim the right to the exclusive use of steam as a motive power for the purpose of producing such effects."

But this very question has been decided in a case precisely similar to this. Charles Goodyear, in his patent of 1849, described a compound of rubber and sulphur. He named no other gum. In his reissue No 1085, dated Nov. 25, 1860, he interpolated the words "other vulcanizable gums." To show the similarity of these patents, I give the originals of Nelson and Charles Goodyear, and the reissues of each:

*Charles Goodyear's patent, December* 25, 1849.

"What I claim as my invention, and desire to secure by letters patent, is the curing caoutchouc or India rubber by subjecting it to the action of a high degree of artificial heat, substantially as herein described, and for the purpose specified.

"And I also claim the preparing and curing the compound of India rubber, sulphur, and carbonate, or other salt or oxide of lead, by subjecting the same to the action of artificial heat, substantially as herein described."

*Nelson Goodyear's patent, May* 18, 1851.

"What I do claim as my invention, and desire to secure by letters patent, is the combining of India rubber and sulphur, either with or without shellacs, for making a hard and inflexible substance hitherto unknown, substantially as herein set forth.

"And I also claim the combining of India rubber, sulphur, and magnesia, or lime, or a carbonate or a sulphate of magnesia, or of lime, either with or without shellac, for making a hard and inflexible substance hitherto unknown, substantially as herein set forth."

*Charles Goodyear's reissue, November* 25, 1860.

"What is claimed as the invention of Charles Goodyear, deceased, is subjecting caoutchouc, or India rubber, or *other vulcanizable gums*, mixed with or in the presence of sulphur (whether with or without other ingredients), to the action of heat, for the purpose of affecting its qualities or properties, as described."

*Nelson Goodyear's reissue, May* 18, 1858.

"But what I do claim as the invention of the said Nelson Goodyear, and desire to secure by letters patent, is the combining of sulphur and India rubber, *or other vulcanizable gum*, in proportion substantially as specified, when the same is subjected to a high degree of heat, substantially as specified according to the vulcanizing process of Charles Goodyear, for the purpose of producing a substance or manufacture possessing the properties or qualities substantially such as described; and this I claim whether the said compound of sulphur and gum be or be not mixed with other ingredients, as set forth."

It is no answer to this objection to say that the mention of all other vulcanizable gums is merely a new *subject*, or use, to which the invention is to be applied. It is not a new *use*, but a new *means*.

The invention of Goodyear, reduced to its simplest form, is a compound process consisting of three steps or elements:

1. Sulphur.
2. Rubber.
3. Heat.

No one or two of these is the invention. The union of them all is the means by which the patentee obtains the result.

Now, in the reissue, he proposes to strike out the second member, to wit: rubber, and insert "all other vulcanizable gums." This is a change in the *means*, in the combination, in the very invention itself. It is not a new result of the invention—the result is the same, to wit: a hard product. It is a new way of producing that result, and one not described or hinted at in the original specification; nor, so far as we have any proof, is it the invention of Nelson Goodyear.

This reissued patent of Charles Goodyear coming before Mr. Justice Clifford, in the case of *Goodyear* v. *The Providence Rubber Co.*, a case fiercely contested and ably tried, the learned judge says:

"But another objection is taken to this claim of a very different character. Express terms of the claim make it include not only India rubber, when compounded with sulphur and subjected to a high degree of artificial heat, but *all other vulcanizable gums*, whether with or without other ingredients. Nothing of the kind is described in any one of the patents granted to the original inventor, not even in the patent to which the claim is annexed.

"Under the circumstances, I am of opinion that the claim of this patent is broader than the invention of the original patentee, and, consequently, that it is void, because it is not for the same invention as the patent which was surrendered as the foundation of the reissue. *O'Reilly* v. *Morse*, 15 How. 112; *Batten* v. *Taggart*, 17 How. 83; *Burr* v. *Duryee*, 1 Wall. (S. C.) p. 531; *Leroy* v. *Tatham*, 14 How. 175."

See *Goodyear* v. *Providence Rubber Co.*, 2 Fisher, 499.

This authority is decisive of this point, so far as authority can be of any value, and it must be overruled or followed.

It is so obviously in accordance with the later deliverances of the supreme and circuit courts upon this vexed question of reissues, that I apprehend that it will recommend itself to the careful consideration of the Court in the present case.

This point is raised under the following paragraph of the answer :

"This defendant, further answering, on information and belief, says that said original patent was surrendered, and said reissues obtained, for the purpose of including and embracing in said reissued patents, matters, and things, and inventions not included, and embraced, and described in said original patent, and that said reissues do include and embrace matters and things, and inventions not included, and described, and contained in said original patent, and of which the said Nelson Goodyear was not the first and original inventor, and are therefore void."

It was not made, argued, or passed upon by Judge Nelson, in either of the cases tried before him.

See *Goodyear* v. *New York Gutta Percha Co.*, 2 Fisher, 312.
*Goodyear* v. *Wait*, pamphlet.

The effect of a decision upon this point, adverse to the patentee, would at most compel him to reissue again, and be more modest in his claims. To sustain such claims in the face of such objection would be to throw open the door for interpolation *ad libitum.*

II. *The reissues were improperly granted.*

One for a process producing inevitably a certain product, and the other for the same product as produced by the process. Such patents could not be granted to separate individuals. How can they be separately granted to the same individual ? If he could receive them, he could dispose of them separately. Would not that be a double sale of the same invention? A new way of making an old thing is doubtless patentable, but not the means, and then the old thing *as made by the new way.*

III. *Infringement is not proved.*

"The burden of the proof on the issue of infringement is upon the plaintiffs. They charge infringement, which is a wrongful act in the nature of a trespass, and, inasmuch as no one is presumed to do wrong, the rule is, that he who alleges that another has committed a wrongful act, must prove it."

Clifford, J., *Union Sugar Ref.* v. *Matthiessen*, 2 Fisher.

The defendants are not infringers because they *buy* the soft rubber compound, and the proof of the complainants, with a single exception, is confined to such purchases. The thing purchased was soft rubber called by certain names. The vendors did not know its composition; and it is not traced to any one who does. General proof that rubber of certain names was made in a certain way, does not prove that the rubber bought by the defendants, though called by such names, was made in the same way.

In Chicago the only proof offered is that of the defendants themselves, who append certain pieces of soft rubber, the composition of which they say is unknown to them, and the commercial names of which they do not give. The composition of this rubber might have been proved by analysis, and it might have been traced to the vendors. It was the duty of complainants to do this, or at all events to prove that this rubber was their rubber. They do not attempt this. All that they do is to attempt to prove a negative, and to ask the Court to find its matter of fact, without evidence, either that the rubber exhibited by the defendants contains sulphur in certain proportions, or that the defendants must infringe because the complainants are entitled to all rubber that will vulcanize, no matter what may be its ingredients or their proportions.

The bill having called for an answer under oath, the defendants have answered under oath denying infringement. The answer can only be overcome by testimony equivalent to the evidence of two witnesses. 3 Green. Ev. 250.

(After a critical examination of the ingredients and proportions of the Simpson rubber the argument proceeds as follows.—Ed.)

"But where does Goodyear get the right to claim four ounces of sulphur to the pound of rubber? *Charles* Goodyear, whose patent is now public property, described his compound as consisting of five parts sulphur to twenty-five parts of rubber; five to twenty-five is one-fifth sulphur, which in one pound would be $3\frac{1}{5}$ ounces. Nelson Goodyear in his original patent, says:

"The indispensable ingredients used in my composition are caoutchouc and sulphur; *and when only these two ingredients are used*, the best proportion will be about *equal parts by weight of each of them, indeed a much less proportion of sulphur will not suffice.* But though the combination of so large a proportion of sulphur with the caoutchouc will produce when cured a hard substance, a still better result will be obtained by the introduction of magnesia, lime, carbonate of magnesia or lime, or sulphate of magnesia or lime, into the composition, in which case the following proportion will be found a highly advantageous one, viz.: One pound of caoutchouc, half a pound of sulphur, and half a pound of magnesia or lime, or carbonate of magnesia or lime, or sulphate of magnesia or lime. The proportions specified in both of these compounds may be considerably varied without materially changing the result; but in no case will a much less quantity of sulphur than four ounces to every pound of caoutchouc be sufficient, in which respect, particularly, my compounds differ very essentially from every other composition of India rubber in use."

Now, beyond all question, the obvious meaning of this is, that when you use rubber and sulphur only, you must use them in equal parts, *i. e.*, 16 oz. of sulphur to 16 oz. of rubber, and a much less proportion would not suffice.

But this proportion may be modified by introducing other ingredients named, so that less sulphur may be used, but in *no* case (not in *either* case), that is, not even by the use of lime and magnesia, can less than four ounces of sulphur be used.

Therefore, when Goodyear claims as low as four ounces, or much less than sixteen ounces of sulphur, he claims it only in combination with other ingredients, and as to them he can claim only those named in his specification, or their chemical equivalents. Neither the complainants, nor their learned chemists, have yet been sufficiently daring to claim that linseed oil and gum benzoin are the equivalents of carbonate of lime or sulphate of magnesia.

The reissues state this matter of proportion very ambiguously, and with intention to deceive; but they do not contradict or correct the specification of the original, and must be construed in the light of the positive statement in the original patent. Compared with the original patent, the Simpson rubber does not, in any form or proportion named in his patent, even as construed or analyzed by complainant's witnesses, infringe the Nelson Goodyear patent, and if the reissues are broader than the original in this respect, they are void.

IV. *The open and notorious purchase and use of soft rubber by the Dentists of this vicinity, for the purpose of making vulcanized plates, is a bar to a proceeding in equity for an injunction. The only remedy of complainants, if they have any, is at law.*

But one suit was ever brought here, to wit: The one against Toland, and that was not litigated. There is no proof that any one was licensed, except Taft and the Ohio Dental College, to both of whom licenses were presented. It is claimed that others are named in the "Vulcanite," but the total does not exceed a half a dozen, and it will hardly be claimed that lists in the publication of complainants are proof of licenses. But both the suit and the licenses were over five years ago. If they show anything, they show the fact that the use of the rubber plates in this vicinity was known to the complainants, and yet for five years not one step has been taken to vindicate what they are now pleased to call their rights. The label was a mere sham, and the testimony shows that it was never regarded.

Judge Story says, in *Wyeth* v. *Stone*, 1 Story, 282:

"I agree that it is quite competent for a patentee at any time, by overt acts or by express dedication, to abandon or surrender to the public, for their use, all the rights secured by his patent, if such is his pleasure, clearly and deliberately expressed. So if, for a series of years, the patentee acquiesces without objection in the known public use by others of his invention, or stands by and encourages such use, such conduct will afford a very strong presumption of such an actual abandonment or surrender. *A fortiori*, the doctrine will apply to a case where the patentee has openly encouraged or silently acquiesced in such use by the very defendants whom he afterward seeks to prohibit by injunction from any further use; for, in this way, he may not only mislead them into expenses, or acts or contracts against which they might otherwise have guarded themselves, but his conduct operates as a surprise, if not as a fraud upon them. At all events, if such a defense were not a complete defense at law, in a suit for any infringement of the patent, it would certainly furnish a clear and satisfactory ground why a court of equity should not interfere, either to grant an injunction, or to protect the patentee, or to give any other relief. This doctrine is fully recognized in *Rundell* v. *Murray*, Jacobs' R. 311, 316, and *Saunders* v. *Smith*, 3 Milne & Craig, 711, 728, 730, 735. But if there were no authority on the point, I should not have the slightest difficulty in asserting the doctrine, as found in the very nature and character of the jurisdiction exercised by courts of equity on this and other analogous subjects."

Judge Drummond says, in *Goodyear* v. *Honsinger*, Mss.:

"I adverted, during the argument, to an inference which might be drawn from the conduct of a patentee—from the conduct of the representatives of the patentee in this case—selling licenses to some parties, and allowing other parties to go on and use the very thing which was licensed to some, without interruption and without objection. It was unfair, and is always unfair, to those who are licensed to use the particular article or method, under letters patent, to allow others to use what the licensees have a right, alone, to use under the license."

And, finally, this Court said, in this very case, when heard upon the motion for a preliminary injunction:

"The Dentists have been in the habit of using it for a number of years, and until lately without any question as to their right to do so. They have acted on the supposition that the owners of the patent had acquiesced in the use of the article for Dental purposes; and they resent any prosecution against the Dentists for infringement. The only question that can possibly arise is that of infringement. That I have not pretended to examine. It will, undoubtedly, be an interesting question, whether the use of this article, under the circumstances connected with the case, is an infringement of the Goodyear patent. If that question should ever come fully before the Court, it will be the duty of the Court to investigate it fully, and endeavor to arrive at a satisfactory conclusion. There may be some circumstances, possibly, that will justify the Dentists in the use of this article. It appears that what is called the Hard Rubber Company were in the habit for years of selling to Dentists their material, of which this vulcanite is made, and in connection with it, selling also, the machinery necessary to manufacture it for Dental purposes. It will be claimed that here was acquiescence on the part of the owners of the patent in the use of this article by Dentists, for Dental purposes, that released them from liability as infringers."

S. S. FISHER.

